# Changes in NR2ab Antibody Levels in the Blood of Patients Depending on their Condition: Acute or Chronic Cerebral Ischaemia

**DOI:** 10.15388/Amed.2026.33.1.8

**Published:** 2026-07-22

**Authors:** Zauresh Akhmetzhanova, Altynshash Jaxybayeva, Aliya Imanova, Vitaliy Kamkhen, Serik Akshulakov

**Affiliations:** 1 Department of Neurology, Astana Medical University, Astana, Republic of Kazakhstan E-mail: ORCID ID; 2 Department of Neurology, Astana Medical University, Astana, Republic of Kazakhstan; Department of Neurology, Asfendiyarov Kazakh National Medical University, Almaty, Republic of Kazakhstan E-mail: ORCID ID; 3 Stroke Center, Astana Multidisciplinary City Hospital No. 2, Astana, Republic of Kazakhstan E-mail: ORCID ID; 4 Department of Epidemiology, Biostatistics and Evidence-Based Medicine, Al-Farabi Kazakh National University, Almaty, Republic of Kazakhstan E-mail: ORCID iD; 5 National Center of Neurosurgery, Astana, Republic of Kazakhstan E-mail: ORCID ID

**Keywords:** stroke, biomarkers, neuron, NMDA receptors, cerebrovascular disease, logistic regression, insultas, biožymenys, neuronas, NMDA receptoriai, smegenų kraujagyslių liga, logistinė regresija

## Abstract

**Aim:**

The study aimed to determine the efficacy of antibodies to the NR2ab subunits of the N-methyl-D-aspartate (NMDA) receptor (NR2ab) as a biomarker for the differential diagnosis of acute and chronic cerebral ischaemia (CCI) among patients in Kazakhstan.

**Materials and methods:**

The study involved 52 patients with CCI classified as second-degree according to the Fazekas scale and 47 patients with acute ischaemic stroke (AIS), rated at 16–20 on the National Institutes of Health Stroke Scale (NIHSS), who were hospitalised in stroke centres within 24 hours of the first symptoms of stroke and confirmed by magnetic resonance imaging. Serum samples were collected to determine NR2ab antibody levels.

**Results:**

The results of the study showed that the mean level of NR2ab in patients with CCI was 1.42 ng/ml (95% CI: 1.2–1.6), while in patients with AIS it was 1.99 ng/ml (95% CI: 1.7–2.3). Statistical analysis revealed a significant difference between the groups (*p*=0.003), and logistic regression determined the prognostic significance of NR2ab with a coefficient of 1.885. The *Receiver Operating Characteristic* (ROC) curve analysis showed a diagnostic accuracy of 0.675, which indicates the prospects of using this biomarker to detect acute cerebral ischaemia.

**Conclusions:**

The results confirm that determining the level of NR2ab can be a useful tool for a timely diagnosis and selection of adequate treatment, especially in conditions of limited access to neuroimaging. Incorporation of this method into the standard diagnostic process can improve the efficiency of detecting ischaemic disorders and optimise patient care.

## Abstract (it)

**Tikslas:**

Tyrimo tikslas buvo įvertinti antikūnų prieš N-metil-D-aspartato (NMDA) receptoriaus NR2ab subvienetus (NR2ab) veiksmingumą kaip biožymenį ūminės ir lėtinės smegenų išemijos (CCI) diferencinei diagnostikai pacientams Kazachstane.

**Medžiagos ir metodai:**

Tyrime dalyvavo 52 pacientai, sergantys lėtine smegenų išemija, pagal Fazekaso skalę klasifikuojama kaip antro laipsnio, ir 47 pacientai, patyrę ūminį išeminį insultą (AIS), kurių būklė pagal Nacionalinių sveikatos institutų insulto skalę (NIHSS) buvo įvertinta 16–20 balų. Jie buvo hospitalizuoti insulto centruose per 24 valandas nuo pirmųjų insulto simptomų atsiradimo ir patvirtintų atlikus magnetinio rezonanso tomografiją. Serumo mėginiai surinkti NR2ab antikūnų lygiui nustatyti.

**Rezultatas:**

Tyrimo rezultatai parodė, kad vidutinis NR2ab antikūnų lygis pacientų, sergančių CCI, buvo 1,42 ng/ml (95 % PI: 1,2–1,6), o pacientų, sergančių AIS, – 1,99 ng/ml (95 % PI: 1,7–2,3). Statistinė analizė atskleidė reikšmingą skirtumą tarp grupių (p = 0,003), o logistinės regresijos metodu nustatytas NR2ab antikūnų prognostinės reikšmės koeficientas 1,885. ROC kreivės analizė parodė 0,675 diagnostinį tikslumą, kuris rodo šio biožymens naudojimo ūminei smegenų išemijai nustatyti perspektyvas.

**Išvados:**

Rezultatai patvirtina, kad NR2ab antikūnų lygio nustatymas gali būti naudinga priemonė laiku diagnozuoti ligą ir parinkti tinkamą gydymą, ypač esant ribotai prieigai prie neurovaizdinimo. Įtraukus šį metodą į standartinį diagnostikos procesą, galima pagerinti išeminių sutrikimų nustatymo efektyvumą ir optimizuoti pacientų priežiūrą.

## Introduction

Cerebral ischaemia, which occurs as a result of acute or chronic cerebrovascular accident, is a complex process accompanied by energy deficit, ion imbalance, oxidative stress and inflammation. It is one of the main factors in the development of neurological disability and high mortality among patients with cerebrovascular disease. Acute ischaemia, and, in particular, stroke, requires urgent diagnosis and treatment due to the risk of irreversible neuronal damage. Chronic ischaemia, which develops gradually against the background of decreased cerebral blood flow, leads to cognitive impairment and encephalopathy, worsening the quality of life of patients. Specific biomarkers are needed for the differential diagnosis of these conditions, and NR2ab antibodies represent a promising area.

An increase in the level of NR2ab in the blood is associated with a breakdown of the blood-brain barrier and neuronal death during ischaemia. In patients with acute ischaemia, there is a rapid increase in the concentration of NR2ab, while in chronic ischaemia, the dynamics are different. Understanding these differences may help clarify the pathogenesis of ischaemia and develop new approaches to diagnosis and treatment. Studying the level of NR2ab in the blood of patients with cerebral ischaemia is an important task that contributes to the research on the pathophysiological mechanisms of neural damage and to propose diagnostic algorithms that will optimise treatment and improve the effectiveness of medical care for patients with cerebrovascular disease.

In modern neurology, the problem of differential diagnosis of various forms of cerebral ischaemia, especially acute and chronic, remains relevant due to the difficulty of establishing the mechanisms of the pathological process, including the role of autoimmune reactions associated with antibodies to NMDA receptors and their components, such as NR2ab. Studies by Tuncar et al. [1] showed that an increase in the level of NR2ab peptides in patients with AIS can be useful for diagnosis, prognosis and assessment of coma indicators. However, the question of the prognostic significance of NR2ab antibody levels in CCI remains relevant. The analysis of blood biomarkers to distinguish between ischemic stroke and intracerebral haemorrhage, conducted by Bhatia et al. [2], demonstrated the prospects of using biomarkers in neurology, but was limited to acute strokes, leaving the diagnosis of chronic ischaemia poorly understood. The study by Ponomarev et al. [3] showed that an increase in the level of NR2ab to NMDA receptors can be a marker of ischaemic damage but did not address differences in antibody levels depending on the stage and duration of the ischaemic process. Thus, further investigation of changes in NR2ab antibody levels depending on the type of ischaemia (acute or chronic) is required.

The studies by Wang et al. [4] and Paolucci et al. [5] investigated the role of antibodies in the pathogenesis of cerebrovascular diseases. The first study showed that antibodies to NR2ab receptors in neuropsychiatric lupus damage the blood-brain barrier, facilitating the penetration of pathological agents into the brain and neuronal damage. The second study found an association of NR2ab to neuronal surface antigens with the severity and prognosis of acute stroke. Both studies highlight the need to further investigate the mechanisms of action of NR2ab in the context of ischaemia and their potential as biomarkers for diagnosis and therapy.

Hadjiagapiou et al. [6] studied NR2ab antibodies, demonstrating the potential of biomarkers for detecting cerebrovascular damage, given their association with neuronal damage. Kuo et al. [7] analysed the role of endogenous activation of the Nrf2/HO-1 axis in ischaemia, which modulates microglial polarisation and limits brain damage, emphasising the importance of antioxidant and anti-inflammatory mechanisms in the pathogenesis of ischaemia. These studies confirm the importance of immunological and molecular processes in the progression of cerebrovascular disease.

Cerebral ischaemia is accompanied with complex pathophysiological processes, in which, neuronal damage, blood-brain barrier disruption and activation of the immune response play a key role [8–10]. NR2ab antibodies are becoming an important marker of these changes, as their blood levels correlate with the severity of the ischaemic process. As noted by Dambinova et al. [11], an increase in the level of NR2ab indicates active neuronal damage due to hypoxia and energy deficit, which is therefore a highly sensitive diagnostic method. Determination of these antibodies is also important for assessing the effectiveness of therapeutic interventions, especially in acute forms of cerebral ischaemia.

Pan et al. [12] show that the activation of autoantibodies to NMDA receptors, especially NR1, is associated with chronic stress and traumatic brain injury. Although the focus is on NR1 antibodies, the results confirm the importance of further studying NR2ab in ischaemic brain damage. The study by Dobrynina et al. [13] complements these data by demonstrating that antibodies to NR2ab are an early marker of cerebral microangiopathy in chronic ischaemia, revealing a relationship between the level of antibodies and the degree of damage to small vessels in the brain. This is especially important for the prediction of cognitive impairment, emphasising its potential as an indicator of ischaemic progression and its consequences. Thus, the study of NR2ab can become a key component of the differential diagnosis of acute and chronic ischaemia, improving the accuracy of prognosis and treatment approaches.

Despite a considerable amount of research on the role of NR2ab antibodies in cerebrovascular pathology, the issues of their dynamics in various forms of cerebral ischaemia remain insufficiently studied. This is especially relevant when comparing changes in antibody levels in acute ischaemia, which is accompanied by rapid neuronal damage, and chronic ischaemia, which is characterised by a gradual progression of the pathological process. Most studies focus mainly on acute conditions, while chronic forms remain insufficiently studied. The study aimed to analyse changes in the level of NR2ab in the patients’ blood depending on the type of cerebral ischaemia to improve differential diagnosis and develop individualised therapeutic approaches.

## Materials and Methods

The study was conducted after obtaining approval from the Institutional Ethics Committee of Astana Medical University (Protocol No. 4/11 of 22 December 2022). The study group was formed from September 2022 to June 2023, involving both outpatients and patients hospitalised in stroke centres. The study was conducted following international standards, including the recommendations of the World Health Organisation, the European Stroke Organisation, and the American Heart Association. The study complied with the Declaration of Helsinki. All patients provided written informed consent to participate in the study.

The study included the first group (Group 1), consisting of 52 patients diagnosed with CCI. These patients were selected from more than 200 people undergoing outpatient care in accordance with our clearly defined inclusion and exclusion criteria. According to the International Classification of Diseases, 10^th^ Revision (ICD-10) [14], a standardised system for coding diseases and health conditions globally for clinical, research, and statistical purposes, the diagnosis of CCI was confirmed based on clinical observation and corresponded to I67.8. The Fazekas scale [15], a visual rating system frequently used to assess the severity of white matter hyperintensities in the brain, ranges from ‘0’ (no lesions) to ‘3’ (severe confluent lesions). Brain *Magnetic Resonance Imaging* (MRI) was performed by using a 1.5-Tesla scanner (Philips Healthcare) and revealed grade 2 white matter lesions according to the Fazekas scale.

The second group (Group 2) included 47 patients who were admitted to Astana City Stroke Centre on an emergency basis within the first 24 hours of the onset of symptoms corresponding to the first-ever case of AIS. With an NIHSS score of 16 to 20, the diagnosis of AIS was verified by using the ICD-10 classification (codes I63.3-I63.5) and strict criteria for ischaemia in the middle cerebral artery basin. With scores ranging from ‘0’ (no impairment) to ‘42’ (severe stroke), the NIHSS is a standardised instrument used to evaluate neurological deficits objectively in acute stroke patients. This aids in determining the severity of the stroke and directing treatment [16].

Both study groups used identical inclusion and exclusion criteria to guarantee sample homogeneity. The study exclusively involved individuals with a validated diagnosis of CCI or AIS, corroborated by clinical evaluation and neuroimaging techniques. Patients with previous strokes (ischaemic or haemorrhagic), conditions mimicking stroke (hypoglycaemia, migraine with aura, Todd’s paralysis), and decompensated somatic diseases were excluded. Patients with severe mental or neurological disorders (meningitis, encephalitis, multiple sclerosis, Parkinson’s disease, etc.), pregnancy, chronic alcoholism or substance abuse, a body mass index greater than 40 kg/m^2^, or metal implants that would make MRI impossible were excluded from the study.

The main demographic and clinical characteristics of the patients cover various aspects (Table 1).

**Table 1:** Characteristics of the patient groups

Characteristic	Group 1 (n=52)	Group 2 (n=47)
Males, %, (n)	28.8 (15)	63.8 (30)
Females, %, (n)	71.2 (37)	36.2 (17)
Mean age, years (95% CI)	55.71 (52.41-59.01)	62.78 (59.13-66.44)
Kazakh nationality, %	100	100
Higher education, %	100	100
Arterial hypertension, %	100	100
Diabetes mellitus, %	32.6 (17)	59.5 (28)
Dyslipidaemia, %	88.4 (46)	89.3 (42)
Body mass index, kg/m^2^ (95% CI)	26.44 (25.36-27.52)	26.28 (25.43-27.13)
Active smokers, %, (n)	19.2 (10)	42.5 (20)

*Source:* compiled by the authors

Cognitive function was assessed by using the Montreal Cognitive Assessment (MoCA) [17], a widely used screening instrument designed to detect mild cognitive impairment. The MoCA tests several cognitive areas, such as memory, attention, language, visuospatial skills, executive functions, and orientation. Scores range from 0 to 30, with higher values indicating better cognitive performance.

Emotional state was assessed by using the Hospital Anxiety and Depression Scale (HADS) [18], a self-administered questionnaire commonly used to screen for anxiety and depression in both inpatient and outpatient settings. The HADS consists of 14 items, with 7 assessing anxiety and 7 assessing depression, each scoring from 0 to 3. This structure allows for the categorisation of symptom severity while minimising the influence of physical illness on assessment outcomes.

Both assessments were conducted upon patient admission to establish baseline psychological and cognitive status, serving as a reference point for subsequent monitoring and intervention.

Venous blood in a volume of 5.5 ml was collected by standard venipuncture into serum tubes. The samples were centrifuged at 3,000 rpm for 10 minutes. Although NR2ab remain stable at room temperature for up to three hours, all serum samples were immediately placed in a refrigerator at +2–+8°C until analysis. The level of NR2ab was determined by enzyme-linked immunosorbent assay (ELISA) using a DRD Biotech kit, in strict compliance with the manufacturer’s instructions.

Data analysis was performed by using *Statistical Package for the Social Sciences* (SPSS) Statistics software (IBM Corp., USA). Descriptive statistics were used to calculate the mean values, confidence intervals, as well as the minimum and maximum values. The Mann-Whitney test was used to assess the statistical significance of differences between the groups. Logistic regression was used to analyse prognostic indicators, and ROC curve analysis was used to assess diagnostic accuracy.

## Results

### NR2ab Antibody Levels in Patients with Chronic and Acute Ischaemia

The examination of NR2ab antibody levels in blood serum revealed significant disparities between patients with CCI and those with AIS, suggesting that NR2ab concentration fluctuates based on the clinical stage and severity of the ischaemic process (Table 2).

**Table 2:** Statistical data on the level of NR2ab in blood serum

Metric	Group 1 (CCI, n=52)	Group 2 (AIS, n=47)
Mean value, ng/ml (95% CI)	1.42 (1.2-1.6)	1.99 (1.7-2.3)
Minimum value, ng/ml	0.51	0.45
Maximum value, ng/ml	3.64	5.25
25th percentile, ng/ml	0.77	1.18
50th percentile (median), ng/ml	1.06	1.69
75th percentile, ng/ml	2.01	2.61

*Source:* compiled by the authors

Patients with CCI typically demonstrated diminished NR2ab levels with restricted variability, indicative of a rather stable chronic pathogenic condition. This trend indicates the gradual advancement of neuronal loss commonly seen in CCI and implies a more uniform disease trajectory within this cohort.

Conversely, patients with AIS exhibited significantly elevated NR2ab levels and a broader distribution. This heightened variability signifies the diverse and fluctuating characteristics of acute ischaemic injury, which may be influenced by infarct location, degree of neuronal damage, and the existence of concomitant diseases. Increased NR2ab levels in AIS signify heightened NMDA receptor-associated neuronal damage, which is typical of acute cerebral ischaemia.

These data suggest that NR2ab levels correlate with both the severity and the clinical progression of the ischaemic process. Elevated values in AIS indicate increased neuronal damage, while the restricted range in CCI reflects a less severe and more stable diseased state.

The Mann-Whitney test indicated a significant difference in NR2ab levels between the two groups (U=795.5, Z=-2.989, *p*=0.003), thereby confirming the diagnostic utility of NR2ab in distinguishing acute from CCI.

Logistic regression was performed to assess the prognostic significance of NR2ab antibody levels in distinguishing between acute and CCI. In this model, NR2ab values were considered as a predictor affecting the probability of diagnosing acute ischaemia (Table 3).

**Table 3:** Results of logistic regression assessing the prognostic significance of NR2ab antibody levels for AIS

	B	Wald	Sig.	Exp(B)	95% CI for Exp(B)	
					Lower	Upper
NR2ab antibody values	0.634	6.974	0.008	1.885	1.178	3.017
Constant	-1.160	6.871	0.009	0.313		

*Note*. The following abbreviations were used: B – regression coefficient; Wald – coefficient significance criterion; Sig. (Significance) – p-value; Exp(B) – odds ratio; 95% C.I. (Confidence Interval) for Exp(B) – confidence interval for the Exp(B) value. *Source:* compiled by the authors

The results demonstrated a clear association between elevated NR2ab levels and an increased probability of AIS. A one-unit increase in NR2ab concentration was associated with an approximately 1.9-fold increase in the likelihood of acute ischaemia, thus indicating that NR2ab has prognostic relevance in identifying acute pathological conditions.

In addition to its utility for group-level differentiation, NR2ab also showed relevance in individual clinical trajectories. Several patients initially classified within the CCI group exhibited NR2ab levels exceeding 3.0 ng/ml. Subsequent clinical follow-up revealed that these patients developed transient ischaemic attacks (TIAs) shortly after blood sampling, suggesting that abrupt increases in NR2ab among patients with chronic ischaemia may reflect disease instability or an impending acute ischaemic event.

A TIA is defined as a transient episode of neurological dysfunction caused by focal cerebral ischaemia without evidence of acute infarction on neuroimaging and is recognised as a strong predictor of a subsequent ischaemic stroke, particularly within the first 48–72 hours following the symptom onset [19]. The observed temporal association between elevated NR2ab levels and the occurrence of TIA supports the hypothesis that subclinical neuronal injury and NMDA receptor-mediated excitotoxic processes may precede overt clinical deterioration.

Overall, these findings indicate that, even in patients with clinically stable CCI, a marked increase in NR2ab antibody levels may signal an unstable pathological state associated with an increased short-term risk of acute ischaemic events. Consequently, NR2ab seems to be a promising biomarker for evaluating the severity and progression of the ischaemic process and may enhance risk stratification for TIA and AIS. Further studies are required to establish clinically relevant threshold values of NR2ab for the early identification of patients at high risk of acute complications.

### Prognostic Significance of NR2ab for Differential Diagnosis

The analysis of NR2ab antibody levels in patients with CCI and AIS showed a relationship between the concentration of this biomarker and the severity of clinical manifestations. In patients with AIS, higher levels of NR2ab were associated with severe neurological symptoms, such as cognitive impairment, emotional instability and significant motor function limitation. This suggests a pathophysiological role of NR2ab in the progression of ischaemic brain damage.

Patients with NR2ab levels above 3.0 ng/ml demonstrated severe acute ischaemia, which corresponded to the NIHSS score (16–20 points). In these cases, the clinical picture included significant cognitive impairment: impaired short-term memory, along with decreased ability to concentrate and perform executive functions. The emotional state of these patients was characterised by increased anxiety and depression, which was confirmed by the HADS and MoCA scales. In addition, motor disorders were manifested in the form of hemiparesis, coordination disorders and a significant decrease in the ability to perform independent care and movement.

In patients with CCI whose NR2ab levels were below 3.0 ng/ml, neurological manifestations were less pronounced. The clinical picture included a gradual slowing of cognitive processes, mild forgetfulness and moderate emotional lability. Patients with intermediate levels of NR2ab (2.5–3.0 ng/ml), especially in the group with CCI, showed a tendency to worsen their condition under the influence of concomitant factors, such as hypertensive crises. This indicates a potential role of NR2ab in predicting the risk of developing TIA or progression to acute conditions.

Logistic regression confirmed the significance of NR2ab as a predictor for the differential diagnosis between CCI and AIS. The research indicated that elevated NR2ab concentrations correlated with an increased likelihood of acute ischaemia, hence affirming its diagnostic significance. The probability estimates in Table 4 exemplify this tendency and facilitate the distinction between chronic and acute diseases without reiterating numerical data throughout the text.

**Table 4:** NR2ab level as a prognostic indicator of acute ischaemia

NR2ab level (pg/ml)	Probability of AIS diagnosis (%)
2.0	52.7
3.0	67.7
4.0	79.8

*Source:* compiled by the authors

The data demonstrate that NR2ab levels are a reliable prognostic indicator that can effectively distinguish between acute and CCI. An increase in this biomarker reflects intensification of the ischaemic process rather than isolated laboratory variation. This also indicates that NR2AB can be integrated into clinical practice with the objective to assess the risk of acute ischaemic events, providing early intervention and personalised treatment approaches. In patients with CCI whose NR2ab levels approach clinically relevant thresholds, dynamic monitoring may be warranted to ensure timely detection of deterioration.

An increase in NR2ab concentration in patients with acute ischaemia correlates with the extent of brain damage, which confirms the importance of this biomarker as an indicator of the severity of the process. High NR2ab values may indicate the activity of autoimmune reactions and the extent of ischaemic damage, which is important not only for early diagnosis but also for predicting the course of the disease. In patients with CCI whose NR2ab levels are approaching thresholds, it is necessary to ensure dynamic monitoring to detect deterioration promptly.

**Figure 1:**
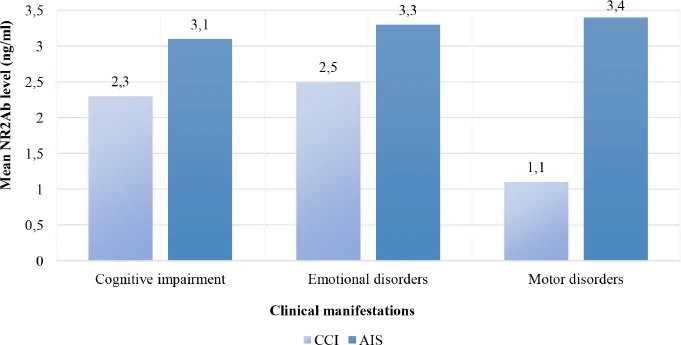
NR2AB levels in patients with CCI and AIS, depending on the type of clinical manifestations *Source:* compiled by the authors

The study of the relationship between the level of NR2ab and clinical manifestations (cognitive, emotional, and motor impairment) was used to assess the specificity of pathological changes in the groups of patients with CCI and AIS (Figure 1).

The analysis of the relationship between NR2ab levels and clinical symptoms demonstrates that an increase in the concentration of this biomarker is closely associated with a deterioration in patients’ condition, especially in cases of acute ischaemia. This may indicate the pathophysiological role of NR2ab in ischaemia-induced neurodegenerative processes. High values of NR2ab in patients with AIS reflect the intensity of brain tissue damage and the activity of the autoimmune component of the pathology, which can be used for early differentiation between chronic and acute ischaemia. Patients with CCI whose NR2ab levels were close to the threshold (3.0 ng/ml) require close monitoring, as they are at risk of acute events. This makes it possible to use NR2ab as a prognostic biomarker to assess the likelihood of transition from chronic to acute ischaemic conditions.

The results of the study demonstrate that an increase in NR2ab levels is accompanied by a deterioration in the neurological status of patients and the severity of the ischaemic process. This highlights the importance of NR2ab as a biomarker for differential diagnosis and risk assessment in patients with cerebral ischaemia. Further studies will help to clarify its clinical value and contribute to the improvement of therapeutic approaches.

### ROC Analysis of NR2ab Diagnostic Accuracy

To evaluate the diagnostic accuracy of NR2ab antibody levels in distinguishing between CCI and acute cerebral ischaemia, we used ROC analysis, which is a widely recognised method for assessing the prognostic efficiency of biomarkers. This method was used to assess the sensitivity and specificity of a biomarker at different threshold levels, which makes it possible to draw conclusions about its diagnostic ability. In the present study, the ROC curve for NR2ab levels was located above the randomness line, which is an important indicator of the diagnostic value of this marker. The shape of the curve indicates a moderate diagnostic efficiency, which meets the needs of clinical practice.

One of the key indicators of ROC analysis is the area under the ROC curve (AUC), which reflects the overall diagnostic accuracy of the marker. In this study, the AUC value for NR2ab was 0.675, which corresponds to an acceptable level of accuracy. AUC ranging from 0.65 to 0.75 indicates moderate diagnostic performance. This can be used to consider NR2ab as a promising biomarker for the auxiliary AIS diagnosis, especially in conditions where access to modern neuroimaging methods, such as MRI, is limited or outright impossible.

The calculation of the sensitivity and specificity of the biomarker at different thresholds showed that, at the NR2ab level of 2.0 ng/ml, the test sensitivity was 52.7%, while the specificity was 57.6%. This means that the biomarker can correctly identify patients with AIS in 52.7% of cases and correctly exclude this disease in 57.6% of cases. However, with an increase in the concentration of NR2ab in the range from 3.0 to 4.0 ng/ml, the diagnostic efficiency increased. In this range, the sensitivity and probability of diagnosis reached 67.7–79.8%. These data indicate that an increase in NR2ab levels correlates with a more severe course of the ischaemic process; therefore, this biomarker can be used not only for diagnosis but also for assessing the risk of complications.

**Figure 2:**
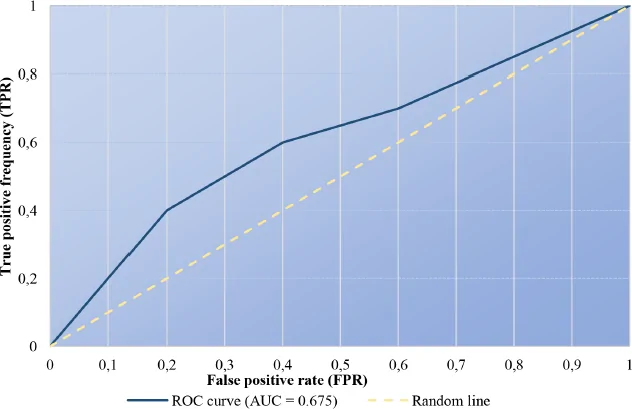
Analysis of the diagnostic accuracy of NR2ab levels using the ROC curve *Source:* compiled by the authors

Figure 2 shows the ROC curve for NR2ab levels, which demonstrates the ratio of sensitivity and specificity of the biomarker in the study groups. The curve deviates significantly from the random line, thus indicating that the marker has diagnostic capability. According to the data obtained, the ROC curve can be used not only to distinguish between patients with CCI and AIS, but also to assess the prognostic value of NR2ab levels in each group. The illustration shows that even in cases where modern neuroimaging methods are not available, this biomarker can help identify patients at high risk of acute ischaemic events. This is especially relevant for the creation of individualised approaches to patient management.

The results obtained highlight the potential of NR2ab as an accessible and easy-to-use biomarker. Its diagnostic capability can be significantly improved by combining it with other biomarkers, such as indicators of metabolic stress, pro-inflammatory cytokines or oxidative stress. In clinical practice, this could become efficient for screening high-risk groups and early detection of acute ischemic conditions.

At the same time, it is worth noting that the diagnostic accuracy of NR2ab remains lower than that of modern imaging methods. However, in cases where these methods are unavailable or limited, the use of NR2ab can provide useful information for clinical decision-making. Further research on combinations of biomarkers and developing algorithms for individualised analysis may increase the diagnostic value of this marker and promote its wider use in clinical practice.

### Comparison of the Diagnostic Efficiency of NR2ab with Modern Methods

Accurate diagnosis of ischaemic brain lesions is a critical task in modern medicine, as ischaemic disorders underlie a significant proportion of neurological diseases, such as stroke, TIA and vascular dementia. Untimely or insufficiently accurate detection of these conditions significantly increases the risk of pathological process progression, including the development of irreversible structural changes in brain tissue, increased oxidative stress with the formation of necrosis foci.

Different methods, including MRI, computed tomography (CT), electroencephalography (EEG) and biomarker detection, have their unique advantages and limitations. In this context, the study of antibodies to the NR2ab subunit offers additional opportunities, providing information that other approaches do not.

MRI is considered the most informative method for diagnosing cerebral ischaemia due to its high spatial resolution. Diffusion-weighted imaging (DWI) methods can detect ischaemic lesions within the first hours after their onset. Perfusion studies (PWI) provide an assessment of blood flow disorders in the brain, which is important for determining the area of ischaemia and the risk of tissue infarction. However, MRI requires high-tech equipment, significant financial costs and qualified personnel, which makes it difficult to use in remote areas or emergency medicine.

CT is a fast and affordable method for detecting acute ischaemic events [20,21]. However, its accuracy in early diagnosis is significantly inferior to MRI, especially in cases of small ischaemic lesions. The sensitivity and specificity of CT are significantly increased with the use of modern perfusion programmes, but, even then, CT does not provide information on the molecular mechanisms of the lesion.

EEG provides data on the functional state of the brain and is useful for monitoring electrical activity. However, this method does not detect structural changes, which limits its diagnostic value in cerebral ischaemia.

Determining the level of biochemical markers in the blood is a promising area that complements traditional neuroimaging methods [22,23]. One of these markers is NR2ab antibodies, which are formed as a result of neuronal destruction and the penetration of degradation products into the bloodstream through the broken blood-brain barrier. Studies show that the level of NR2ab increases in acute ischaemia, reaching peak values in the first 24–48 hours after the onset of the disorder.

Alongside NR2ab, the commonly used biomarkers include C-reactive protein and D-dimers. C-reactive protein reflects systemic inflammation and is associated with vascular risk, whereas D-dimers indicate activation of coagulation and fibrinolysis. However, both markers lack specificity and do not directly reflect neuronal damage, thereby limiting their diagnostic utility in cerebral ischaemia [24].

The NR2ab level is characterised by a sensitivity of 52.7% and a specificity of 57.6%, which indicates a moderate diagnostic accuracy of this method. One of the advantages of the assay is its speed, as the results can be obtained within 4 hours, making it suitable for rapid clinical decision-making. In contrast to neuroimaging methods, NR2ab determination does not depend on the availability of expensive equipment and can be performed in most clinical laboratories using ELISA.

To objectively assess the capabilities of different approaches in the diagnosis of cerebral ischaemia, a comparative analysis of the main methods was performed (Table 5).

**Table 5:** Comparative analysis of the main methods for diagnosing cerebral ischaemia

Method	Sensitivity	Specificity	Time required	Availability	Information about molecular mechanisms
MRI	>90%	>90%	Up to 1 hour	Limited	Absent
CT	70-80%	60-70%	Up to 30 minutes	High	Absent
EEG	40-50%	50-60%	Up to 1 hour	High	Absent
NR2AB	52.7%	57.6%	Up to 4 hours	High	Present
C-reactive protein	40-60%	50-65%	Up to 2 hours	High	Absent
D-dimers	50-70%	55-70%	Up to 2 hours	High	Absent

*Source:* compiled by the authors

The comparative analysis presented in Table 5 suggests that NR2ab antibody testing should be regarded as an adjunctive diagnostic modality rather than a definitive standalone test. Its primary clinical significance resides in circumstances where neuroimaging is inaccessible, delayed, or yields inconclusive results, especially during the initial phases of cerebral ischaemia. Elevated NR2ab levels may assist in identifying patients at heightened risk of TIA or AIS who necessitate more intensive monitoring or additional diagnostic assessment.

From an operational standpoint, NR2ab testing can be incorporated into existing diagnostic protocols as a supplementary second-line evaluation in conjunction with clinical assessment and conventional neuroimaging. In emergency or resource-constrained environments, NR2ab measurement may facilitate early risk stratification while awaiting definitive imaging [25–27]. In patients with CCI, elevated NR2ab levels may signify clinical instability and warrant enhanced monitoring [28].

While the present study validates the diagnostic and prognostic relevance of NR2ab, it does not facilitate direct conclusions regarding their impact on mortality or long-term functional outcomes. Nevertheless, earlier recognition of high-risk patients may enable more prompt diagnostic clarification and intervention. Additional prospective studies are necessary to establish whether NR2ab-guided strategies enhance clinical outcomes.

## Discussion

The results of the study confirmed that the level of NR2ab antibodies in blood serum is a promising biomarker for the differential diagnosis of acute and CCI. The data obtained demonstrated a significant difference between the average NR2ab levels in patients with AIS (1.99 ng/ml) and CCI (1.42 ng/ml), which is confirmed by statistical significance (*p*=0.003). This indicates the potential of NR2ab as an accessible marker for the early detection of ischemic disorders, especially in settings with limited access to modern neuroimaging methods.

These results are in line with the findings of Dambinova et al. [29], demonstrating that an increase in the level of NR2ab is a marker of neuronal damage in ischaemic brain damage. The study noted that NR2ab can be used to monitor the extent of brain tissue damage and predict the effects of ischaemia. On the other hand, some authors, such as Khasanova and Danilova [30], emphasise that the specificity of this biomarker remains insufficiently studied, as an increase in NR2ab levels may be associated with other neurodegenerative processes, such as chronic hypoxia or neurodegenerative diseases. This is consistent with the observations of an increase in NR2ab levels in some patients with chronic ischaemia who have undergone TIA.

The data obtained are also consistent with the studies of Zharkinbekova [31], who studied the pathogenetic mechanisms of CCI and found that the gradual accumulation of NMDA receptor breakdown products can lead to a chronic neuroinflammatory response. The author notes that the NR2ab level can be used to assess the progression of ischaemia and to select treatment tactics. In the present study, NR2ab levels in patients with chronic ischaemia correlated with clinical manifestations, such as cognitive impairment, which may serve as an additional argument in favour of using this marker to monitor patients with chronic ischaemia.

In the context of differential diagnosis, the results of this study partially coincide with the findings of Usmanova and Majhidova [32], who demonstrated that the use of NR2ab in combination with neuroimaging methods, such as MRI, improves diagnostic accuracy and improves the accuracy of assessment of the degree of brain tissue damage. However, the data obtained in the present study indicate that, in conditions of limited access to MRI, the determination of NR2ab levels can be a valuable screening method that can identify patients at high risk of acute ischaemia.

The results with the study of Maestrini et al. [33] confirm the importance of using biomarkers in the early stages of ischaemic brain damage. The study determined that identification of the level of specific antibodies, particularly NR2ab, can be useful for differentiating between TIA and AIS, which is consistent with the data obtained in this study. According to Demchenko [34], the level of NR2ab is considered one of the promising markers of the progression of CCI. The author notes that an increase in the concentration of NR2ab correlates with the deterioration of cognitive function and the progression of the pathological process, which is consistent with the results obtained, demonstrating an increase in the level of NR2ab in patients with more severe clinical manifestations.

The study by Dolmans et al. [35] also confirms the diagnostic value of serological biomarkers, such as NR2ab, for the detection of TIA at the primary level of medical care. The authors highlighted the need to use such biomarkers in combination with clinical data to improve diagnostic accuracy, which is consistent with the results of the present study, which revealed a correlation between NR2ab levels and clinical manifestations.

Tang et al. [36] proved that the combined use of natural compounds such as Astragalus membranaceus and Ligustrazine helps reduce brain damage in ischemia by regulating the NR2B subunit of the N-methyl-D-aspartate receptor and the extracellular signal-regulated kinase/cAMP response element-binding protein pathway (NR2B-ERK/CREB signalling pathway). The data obtained on the role of NR2ab subunits in the development of the ischemic process confirm the pathophysiological significance of NR2ab as a marker of ischemic damage and a potential therapeutic target.

González-García et al. [37] demonstrated that the circulation of autoantibodies to the NR2ab peptide of the NMDA receptor is associated with subclinical brain damage in patients with hypertension and other vascular risk factors. Our results confirm the presence of elevated levels of NR2ab in patients at high risk of developing ischaemic events, which is consistent with the findings of this study.

A systematic review and meta-analysis conducted by Debette and Markus [38] emphasised the clinical significance of MRI white matter hyperintensities as a predictor of cerebrovascular events. The study recommended a combination of neuroimaging methods with biomarker detection to improve prognostic accuracy. The results of the present study are consistent with such approaches, as they revealed a significant increase in NR2ab levels in patients with ischaemic events, which could potentially be used as an additional criterion for diagnosing and monitoring the disease.

The data of the present study are consistent with the findings presented in the study by Wardlaw et al. [39], where the mechanisms of development of sporadic cerebrovascular diseases of small vessels were analysed based on neuroimaging studies. The study noted that damage to small cerebral vessels is the main pathophysiological mechanism of chronic ischaemia, as evidenced by elevated levels of biomarkers of neuronal damage, particularly NR2ab. Detection of these antibodies can provide early diagnosis, complementing neuroimaging data.

The present study determined that elevated levels of NR2ab in the serum of patients with acute cerebral ischaemia are a marker of blood-brain barrier disruption and an indicator of neuronal damage. This is consistent with the results of Wardlaw et al. [40], which indicate that dysfunction of small cerebral vessels is accompanied with an impaired barrier function, which, in turn, leads to the release of specific neuronal proteins into the systemic bloodstream. Thus, elevated levels of NR2ab may indicate progressive small vessel damage, which is important for the differential diagnosis between acute and chronic ischaemia.

The results also confirm the findings of J. Kamtchum-Tatuene and Jickling [41], determining that biomarkers such as NR2ab are denoted by significant potential in the diagnosis of acute ischaemic conditions. The authors noted that elevated levels of NR2ab are observed in patients at high risk of stroke, which is consistent with this study, which established a correlation between antibody levels and the clinical severity of ischaemic brain damage. The revealed tendency to increase NR2ab before the clinical manifestation of ischemia confirms the prognostic significance of this biomarker.

Stanca et al. [42] demonstrated that the level of NR2ab antibodies increases in proportion to the degree of brain tissue damage in acute cerebrovascular disorders. This is consistent with the results obtained, which showed that the highest levels of NR2ab are observed in patients with severe forms of acute ischaemia accompanied by significant neurological disorders. The data obtained confirm the use of NR2ab as a marker of disease severity and a potential tool for monitoring the effectiveness of therapy.

Weissman et al. [43] demonstrated that the level of NR2ab is increased in patients with repeated episodes of TIA, and that it can be used to assess the risk of stroke. The data obtained in the present study on a significant increase in NR2ab in patients with TIA are consistent with the findings of the authors, which emphasises the importance of using NR2ab in screening patients at high risk of stroke.

The results obtained in the present study also partially coincide with the findings of Liang et al. [44], who studied changes in brain white matter by using diffusion tensor imaging in chronic and acute ischaemia. The correlations found between the degree of white matter changes and elevated NR2ab levels indicate that this biomarker may reflect a progressive deterioration in the structural integrity of the brain. The present study also demonstrated that elevated NR2ab levels correlated with the severity of cognitive impairment, which is consistent with the findings on the importance of structural changes for the clinical manifestations of ischaemia.

The present study determined that NR2ab levels in patients with acute cerebral ischaemia reached values of 3.0–4.0 ng/ml, which differentiated between acute versus chronic ischaemia with moderate accuracy. This is consistent with the data of Hooper et al. [45], noting that patients with severe lesions of small cerebral vessels, as identified by the Fazekas scale, had a reduced treatment efficacy and an increased risk of recurrent ischaemic events. The findings of both studies confirm that the use of NR2ab as a biomarker can complement neuroimaging data to assess the risk of adverse outcomes in patients with cerebrovascular disease.

Streifler and Maillard [46] noted that, in patients with leukoaraiosis, the results of thrombolytic therapy are less effective due to structural changes in white matter that reduce the ability of the brain tissue to regenerate. This is consistent with the results obtained, as patients with elevated NR2ab levels and concomitant white matter lesions demonstrated worse functional outcomes, even with adequate baseline therapy. The results of the study by Ryu et al. [47] showed that a greater volume of leukoaraiosis on MRI correlated with worse outcomes in ischaemic stroke, regardless of the treatment used. This is also consistent with the results of the present study, where patients with high levels of NR2ab and severe pathological changes in white matter had a more severe course of acute cerebral ischaemia, as confirmed by the NIHSS scale. The pathophysiological basis for this relationship may be chronic hypoperfusion, which complicates tissue recovery after acute ischemic events.

Arsava et al. [48] determined that the severity of leukoaraiosis on MRI was closely associated with adverse clinical outcomes after ischaemic stroke. The results also confirm this, as patients with high levels of NR2ab and concomitant white matter changes were more likely to have a poor prognosis. Biochemical markers such as NR2ab can be used as an additional tool to assess the severity of the ischaemic process and the risk of adverse outcomes [49,50].

However, there are certain limitations to the use of NR2ab in clinical practice. One of the key aspects is the relatively low specificity of this biomarker at levels below 2.0 ng/ml, which can lead to false-positive results in patients with chronic vascular lesions. However, the data confirm that an increase in NR2ab levels above 3.0 ng/ml is associated with a high probability of acute ischaemic events, thereby rendering this threshold potentially useful for clinical decision-making.

Determination of the level of NR2ab is a promising tool for early diagnosis and prognosis of ischaemic brain disorders. The results demonstrate that NR2ab can be used as a complement to already existing diagnostic methods for more accurate risk stratification in patients with cerebrovascular disease. Further research should be aimed at studying the dynamics of NR2ab levels during treatment and determining their relationship with the functional recovery of patients after acute ischaemia.

## Conclusions

The study determined that the level of NR2AB in the blood serum can serve as an effective biomarker for the differential diagnosis of acute and chronic ischaemic brain damage. The analysis revealed a significant difference between the mean NR2ab levels in patients with different forms of ischaemia: in the acute ischaemia group, the mean level was 1.99 ng/ml (95% CI: 1.7–2.3), while in the chronic ischaemia group it was 1.42 ng/ml (95% CI: 1.2-1.6). These data confirm that NR2ab is a marker capable of reflecting the degree of intensity of the ischaemic process.

The logistic regression analysis showed that a 1 ng/ml increase in NR2AB antibody levels was associated with an 88% increase in the probability of diagnosing acute ischaemia (Exp(B)=1.885; 95% CI: 1.178–3.017; *p*=0.008). This indicates a high prognostic value of this biomarker, which can be used in clinical practice to differentiate between acute and chronic ischaemic processes.

The results demonstrate that the determination of NR2ab levels can become an important part of diagnostic algorithms, especially in conditions of limited access to modern neuroimaging methods such as MRI or CT. The use of this biomarker can significantly reduce the time to diagnosis, ensure early identification of patients at high risk of complications, and improve the quality of therapeutic interventions.

It is recommended to conduct further studies involving larger samples of patients from different regions to increase the representativeness of the data obtained. In addition, it is necessary to study the dynamics of changes in NR2ab levels in patients during treatment, which will help assess the effectiveness of therapeutic interventions.

This study has several limitations. First, the relatively small sample size limits the ability to extrapolate the results to the general population. Second, concomitant pathologies, such as systemic inflammatory or autoimmune processes, may have influenced NR2ab levels, which requires further analysis. In addition, the study focused exclusively on biomarkers, whereas their combined use with neuroimaging methods could provide more accurate results.

A promising area is the development of diagnostic algorithms that combine NR2ab level determination with other biomarkers and clinical indicators. It is also important to establish NR2ab threshold values for identifying patients at high risk of developing complications. In addition, the potential of this biomarker for monitoring treatment efficacy and predicting long-term outcomes of ischaemic brain injury needs to be investigated. Thus, the results of this study confirm the significant potential of NR2ab as an innovative diagnostic tool capable of improving the quality of medical care for patients with cerebrovascular diseases.
